# Green forage and fattening duration differentially modulate cecal microbiome of Wanxi white geese

**DOI:** 10.1371/journal.pone.0204210

**Published:** 2018-09-25

**Authors:** Xingyong Chen, Xue Liu, Yeye Du, Bin Wang, Ning Zhao, Zhaoyu Geng

**Affiliations:** 1 College of Animal Science and Technology, Anhui Agricultural University, Hefei, P.R. China; 2 Anhui Province Key Laboratory of Local Livestock and Poultry Genetic Resource Conservation and Bio-breeding, Anhui Agricultural University, Hefei, P.R. China; 3 Department of Primary Education, Tongcheng Teachers College, Tongcheng, P.R. China; University of Illinois, UNITED STATES

## Abstract

Gut microbial ecology is responsible for fatty acid metabolism in ruminants. The cecal microbiota composition of geese and their adaptation to fiber inclusion and feeding timeswere investigated in this study. A total of 116 Wanxi white geese were randomly selected at 70 days old. Eight geese were subjected to cecal sampling at 70 d of age, and the remaining 108 geese were divided into four groups with three replicates each (9 geese in each replicate). The geese in the four groups were fed 0, 15, 30, and 45% green forage (relative to dry matter), respectively. Three birds from each replicate were selected for cecal sampling at 80, 90, and 100 days old. All samples were subjected to 16S rRNA gene sequencing using the Illumina Ion Personal Genome Machine platform. Bacterial abundance was analyzed using two-way ANOVA analysis, and the relationship between the relative abundance of bacteria (phylum level) and fatty acids was analyzed using acanonical correspondence analysis. Cecal microbiota in geese were mainly composed of *Bacteroidetes* (68.46%), *Firmicutes* (20.04%), and *Proteobacteria* (7.89%). Dietary treatments had no significant effect on the α-diversity indices of the cecal bacterial community (P > 0.05), but a numerical increase occurred with increased fattening duration and green forage inclusion. The *Selenomonadales* order (P = 0.024), *Negativicutes* class (P = 0.026), and *Megamonas* (P = 0.012) and *Oscillospira* (P = 0.042) genera were affected by green forageinclusion level, and microflora abundance was mainly influenced by the fattening duration. Bacteria phyla were mostly set along the line of linolenic acid and oleic acid. Finally, *Bacteroidales* might be an intestinal promoter that improves unsaturated fatty acid synthesis in geese.

## 1. Introduction

Dietary fiber is a major component of plant cell wall, and it consists of both non-starch polysaccharides and non-carbohydrate component. Previous experiments reported that the fiber source in poultry diets plays an important role in gastrointestinal development and, ultimately, energy metabolism [1]. Inefficient and variable responses to dietary fiber result from the absence of digestive enzymes for fiber and the relatively short digestive tract and digestive transition time associated with poultry gastrointestinal tracts [2,3]. Products from ruminants fed high fiber diet display increased bioactive unsaturated fatty acid (UFA)content [4]. Geese are herbivorous poultry that possess a relativelyhighly developed cecum withhemi-cellulose and cellulose digestibility of 41.5% and 17.4%, respectively, aided by the actions of cecal microbiota [5]. The complex composition of cecal microbiota can digest dietary fiber into lower fatty acids (FAs) to make it more dissolvable and absorbable. Thus, the addition of dietary fiber to goose feed couldnot only increasedigestive transit time, but also promote nutrient absorption [6]. Traditionally, geese bred for meat are mainly reared outside withfree access to grass. In modern husbandry, intensified geese production has led to the use of concentrated diets similar to those of broilers and ducks, which caused rapid growth and fat deposition in the abdomen with reduced UFA content [7]. Thus, there is an urgent need to formulate proper green forage asan additional dietary supplement during the geese fattening process.

Research has determined that gut microbial ecology is responsible for FA metabolism in ruminants [8], and several studies of cecal microbiota characteristics of broilers and laying hens were recently reported [3, 9–11]. However, little is known about the composition of goose cecal microbiota and their adaptation to fiber addition and feeding time. In addition, research is needed to clarify whether the gut bacteria in geese are also related to FA synthesis in a manner that is similar to that observed in ruminants. Wanxi white geese, a globally known Chinese indigenous breed, usually undergo fattening between 70 and 100 days old. Therefore, the present study aims to characterize the composition of microbiota in the cecum of Wanxi white geese during fattening with or without inclusion of different ratios of green forage, and to determine the relationship between microbiota and breast FAs composition.

## 2. Materials and methods

All experimental procedures were performed in accordance with guidelines developed by the China Council on Animal Care and Protocols and were approved by the Animal Care and Use Committee of Anhui Agricultural University, China.

### 2.1. Grouping and treatment of geese

A total of 116 male Wanxi white geese, obtained from Anhui Wanxi White Goose Preservation Farm, were raised in a floor system in open-sided houses. All geese were fed a commercial maize-soybean-based diet containing 155 g crude protein/kg feed with a metabolizable energy content of 15.11 to 15.89 MJ/kg feed. At 70 days old, 8 geese were randomly selected, weighed and then for sampling. The left 108 geese were individually weighed, and were randomly allocated to one of the four following green forage treatment groups (three replicates with nine geese per replication), and were treated as indicated: Group 1 was fed a concentrated diet without green forage supplements (white cabbage); Groups 2, 3, and 4 were fed 15, 30, and 45% green forage (relative to dry matter) plus a concentrated diet, respectively (Table 1). Geese were first fed green forage in each day from 8:00 am. Concentrated diet was then provided after green forage been consumed completely. Concentrated diet consumption was recorded in each replicate.

**Table 1:** Nutrient composition of the experimental diets for Wanxi white geese from 70 d to 100 d of age.

chemical composition	percentage of green forage relative to dry matter			
	0	15	30	45
water content %	11.14	12.65	13.78	14.12
ash %	7.11	7.02	6.95	6.82
crude protein %	16.11	15.94	15.72	15.37
crude fat %	1.98	1.92	2.00	1.96
crude fibre %	3.19	5.34	5.69	5.88
calcium %	2.00	1.98	1.96	1.92
potassium %	0.60	0.67	0.72	0.77
metabolisable energy, MJ/kg	15.89	15.42	15.21	15.11

### 2.2. Sample collection and genomic DNA extraction

On day 70, eight geese were randomly selected before being grouped, and were then euthanized for gut sampling after a 12 h fast. Three geese from each replicate were randomly selected at the age of 80, 90, and 100 d, and were then euthanized for gut sampling after fasting for 12 h. Cecal samples were quickly collected and stored at -80 °C before DNA extraction. Goose was first administered a 10 ml 53 °C alcohol directly into the crop via oral gavage. About 30 minutes later after gavage, goose was euthanized by carbon dioxide inhalation and was bled immediately after euthanasia. After the body temperature was completely cooled down, breast muscle (*pectoralis major*) was collected and immediately stored in -20 °C for fatty acid composition analysis.

Total genomic DNA was extracted from 150–200 mg of frozen cecal contents using a QIAamp DNA Stool Mini Kit (QIAGEN, USA). The quality of the extracted genomic DNA was determined using a Qubit 2.0 fluorometer (Invitrogen, Q32866, California, USA). Agarose gel electrophoresis of genomic DNA showed a band with a molecular weight > 10kb and no degradation.

### 2.3. Microbial sequencing

The V3-V4 region of16S rDNA was amplified using a pair of universal primers (334F: CCTACGGGAGGCAGCAG and 519R: ATTACCGCGGCTGCTGG). The 50 μL PCR amplification reaction volume consisted of 2 μL Taq enzyme, 1 μL 10 μM primers, 5 μL genomic DNA (30 ng), and water up to 50 μL. The PCR protocol was as follows: initial denaturation at 98 °Cfor 45 s; eight cycles at 98 °C for 15 s, 57 °C for 30 s, and 72 °C for 60 s; and a final elongation step at 72 °C for 60 s. The amplified PCR products were purified using a Magnetic Stand-96 (Life Technologies, AM10027). Products were then quantified using a Qubit 2.0 fluorometer (Invitrogen, Q32866), and the lengths of the amplified fragments were detected using an Agilent 2100 Bioanalyzer (Agilent, G2939AA). The amplified products were purified using magnetic beads. The concentration was determined using a Qubit2.0, and fragment distribution was detected using an Agilent 2100 Bioanalyzer. The quantified PCR fragments were used to construct libraries using an Ion Plus Fragment Library kit (Life Technologies, USA) according to the manufacturer’s instructions. Library sequencing was performed using an Ion Personal Genome Machine (PGM) system (Life Technology, USA) with an Ion^™^316 Chip and an Ion PGM Sequencing 200 Kit v2 (Life Technology, 4482006), according to the manufacturer’s guidelines.

### 2.4. Fatty acid content and composition analysis

The fatty acid profile was determined after the homogenized fat samples (or dried green cabbage) were defrosted. A 2 g sample was extracted by using chloroform:methanol (2:1, v/v) solution according to the Folch’s method and esterified with methyl alcohol (93%) containing HCL (3%), then applied to DEGS column (DB-WAX, 0.25mm× 0.25μ,) and were detected with FID by gas chromatography (Agilent GC7890A). Proportions of fatty acids are reported as percentages of total fatty acids by mass.

### 2.5. Data analysis

The sequenced Ion PGM raw reads were quality filtered using the FastX Toolkit 0.0.13. Valid reads were then clustered into operational taxonomical units (OTUs) using the Ribosomal Database Project (RDP) classifier method, OTUs were then aligned using the Silva database (http://www.arb-silva.de). OTUs that exhibited at least 97% nucleotide similarity were used for alpha diversity (Shannon and Simpson), richness (ACE and Chao1), Good’s coverage, Venn diagrams, and rarefaction curve analyses using Mothur software (version1.34.0, http://www.mothur.org). OTUs were taxonomically categorized using the naïve Bayesian RDP classifier foundin the Silva database, with a minimum confidence score of 0.8. For downstream analysis, OTUs were filtered by discarding those that comprised fewer than 0.03 of all sequences. To evaluate the α-diversity of the samples, the rarefaction curves of phylogenetic diversity (PD) and a number of observed OTUs were computed using Mothur software. To normalize sequencing depth, the lowest counts among the samples were randomly subsampled in each library 1,000 times, and average values were used to measure diversity indices. The differences between mean values were identified using an analysis of two-way anova test conducted with SAS 9.3 software (SAS Inst. Inc., Cary, NC, USA). Microbial in each taxonomy level that were significantly different among treatment groups were further regressed on green forage supplementation using the following equation:

y=a+bx

Data for fatty acid (FA) content was analyzed by using PROC MIXED of SAS. The statistical model used included green forage, fattening duration and green forage × fattening duration as fixed effects, and geese as random effect. A canonical correspondence analysis (CCA) was used to analyze the relationship between the relative abundance of bacteria (at the phylum level) and FA contents [12].

## 3. Results

### 3.1. Data summary

**Fig 1:**
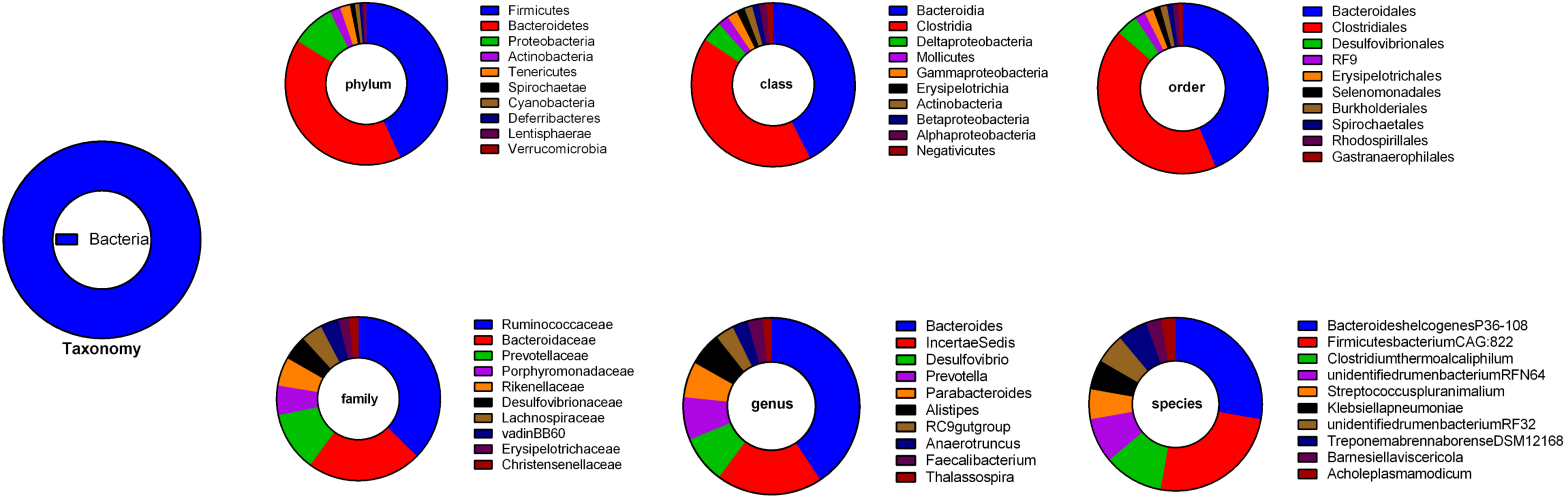
Ten most abundant operational taxonomical units (OTUs) at each level.

A total of 3,724,695 reads from the ceca microbial community were identified after filtering the sequences for quality control, sequencing errors, and chimeras. A total of 2,426,622 valid reads were screened by comparing the paired-end primers with a valid proportion of 63.74%. The total number of reads and valid reads from the original FASTQ files of each sample are presented in S1 Table. There were 9,286 OTUs obtained using the RDP classifier with a 97% confidence rating when compared to the Geese gut microbial Database. Each OTU was then classified based onkingdom, phylum, class, order, family, genus, and species levels with 9,286, 3,952, 3,633, 3,633, 2,604, 1,580, and 10 OTUs in each level, respectively. The most abundant OTUs in each levelare presented in Table 2 and Fig 1.

**Table 2:** The top abundant operational taxonomical units (OTUs) at each level.

Level	Top OUT category description	OUT Numbers
(Taxonomy)	(Bacteria)	9,286
phylum	*Firmicutes*	3,952
class	*Bacteroidia*	3,633
order	*Bacteroidales*	3,633
family	*Ruminococcaceae*	2,604
genus	*Bacteroides*	1,580
species	*Bacteroides helcogenes* P36-108	10

### 3.2. Effects of fattening duration on cecal microbiota

**Fig 2:**
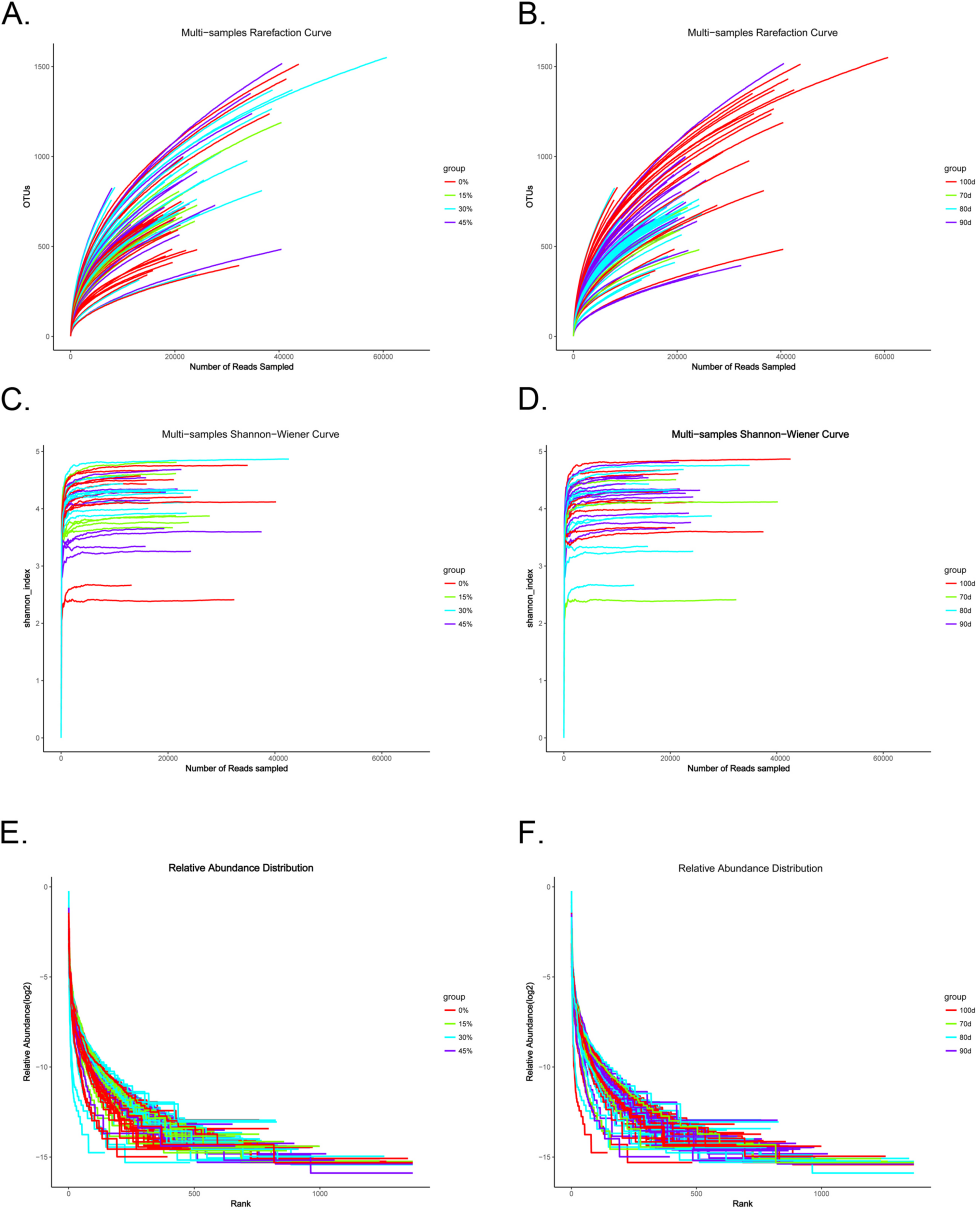
Alpha-diversity indices of the cecal bacterial community. Rarefaction analyses, based on 97% dissimilarity, were used to assess operational taxonomical units (OTUs) among groups with different (A) levels of green forage and (B) fattening duration. Shannon-Wiener analyses among groups with different (C) level of green forage and (D) fattening duration, and the abundance of microbiota among groups with different (E) levels of green forage and (F) fattening duration are shown.

**Fig 3:**
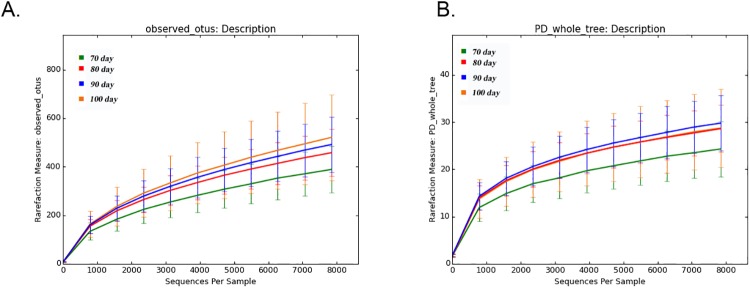
Effects of fattening duration on cecal microbiota diversity and composition. Rarefaction curves calculated at the lowest subsample size of 10,000 sequences per sample indicate the effects of sequencing efforts on the observed number of operational taxonomical units (OTUs) at (A) 97% sequence similarity and (B) phylogenetic diversity (based on the entire tree).

To assess the community α-diversity, the number of observed OTUs (at the 97% level) and PD values were calculated to evaluate whether the sequencing level was adequate for analysis. Rarefaction curves for the observed OTUs, Shannon-Wiener curves, and rank abundance curves approached a plateau, indicating that the sequencing depth was sufficient for the coverage of all OTUs present in the cecal samples (Fig 2). None of the dietary treatments had significant effects on the α-diversity indices of the cecal bacterial community (P > 0.05, Fig 2A–2F). However, α-diversity exhibited a numerical, but not significant, increase with increasing fattening durations. PD values also differed with fattening duration, but the differences were not statistically significant (Fig 3A and 3B). Rarefaction curves for the observed OTUs and PD values approached a plateau, indicating that the sequencing depth was sufficient for the coverage of all OTUs present in the cecal samples.

### 3.3. Effects of green forage supplementation on cecal microbiota

**Fig 4:**
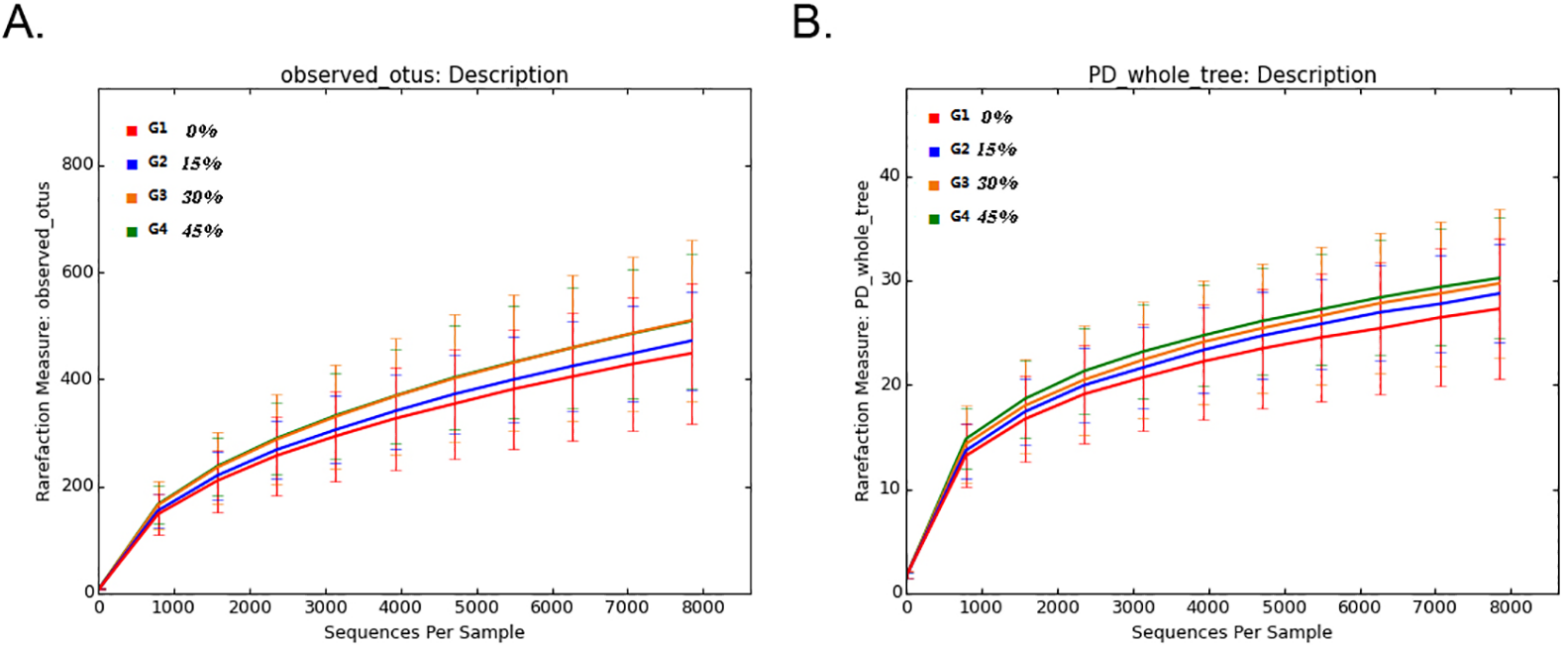
Effects of green forage inclusion on cecal microbiota diversity and composition. Green forage inclusion did not significantly affect the observed number of (A) operational taxonomical units (OTUs) and (B) phylogenetic diversity (based on the entire tree).

Rarefaction curves of 8,000 subsampled reads in the cecum showed comparable numbers of OTUs (at the 97% identity level) for each green forage supplemented group (Fig 4A and 4B), and there were no apparent differences in the rarefaction curves. However, numerical differences were found with regard to the lowest OTUs in cecal samples from geese fed a diet without green forage supplementation as well as the highest OTUs in those from geese fed diets with 30–45% green forage supplementation. Rarefaction for the PD values also exhibited the same trend with the OTU values. Rarefaction curves approached the saturation plateau at a sequencing depth of 2,000 in all sequenced samples.

### 3.4. Effects of fattening duration and green forage treatments on the α-diversity of cecal microbiota

**Fig 5:**
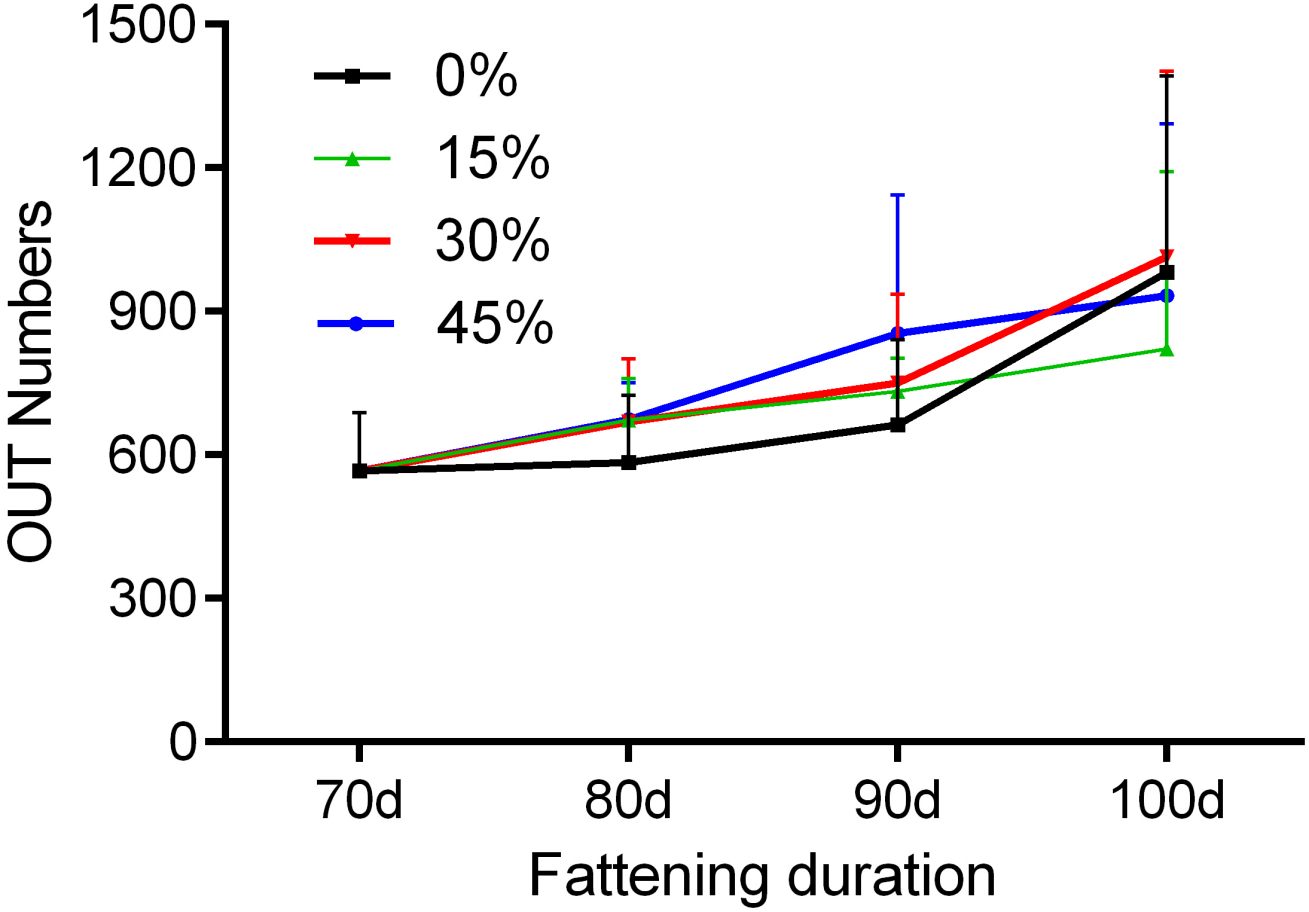
Comparison of cecal microbiota abundance in geese based on fattening durations and green forage inclusion levels. Data represent means ± standard deviation of microbiota based on the fattening duration (time) and green forage inclusion (colored line).

Comparisons ofthe effects of fattening duration and green forage inclusion levels (Fig 5) indicated that the cecal microbial community of geese fed without green forage was significantly different from those of geese fed 15, 30, and 45% green forage (P = 0.001, P < 0.001, and P < 0.001, respectively). Furthermore, before 90 days of age, the cecal microbial community of those fed 15% green forage differed from those fed 30% green forage (P < 0.001). No significant differences were observed between geese fed 30% and 45% green forage (P = 0.598).

### 3.5. Microbial community structure at each taxonomic level

OTUs were taxonomically classified into different levels using the RDP classifier at a 97% confidence threshold. The classification of sequences from samples resulted in 22 phyla, 47 classes, 79 orders, 147 families, and 300 genera (S2 Table and S1–S5 Figs). The relative abundance of OTUs occupying > 1% was used for analyses at different levels (i.e., from phyla to genera) and compared among groups.

At the phylum level (Table 3, S6A Fig), the cecal microbiota community was mainly composed of *Bacteroidetes* (68.46%), *Firmicutes* (20.04%), and *Proteobacteria* (7.89%). No significant difference was detected in the microbiota composition at the phylum level among the green forage-fed groups. However, the percentage of *Bacteroidetes* and *Spirochaetae* in cecal samples of geese differed based on the fattening time (P = 0.041). The percentage of *Bacteroidetes* in cecal samples from geese that were fattened for 10 days was significantly higher than that of geese fattened for 20 or 30 days (P = 0.023 and P = 0.047, respectively). The percentage of *Spirochaetae* in cecal samples of geese before fattening was significantly lower than that of geese fattened for 10 and 20 days (P = 0.043 and P = 0.001, respectively).

**Table 3:** Abundances of the major phylum in each group and fattening time.

Fattening duration	Green forage	*Deferribacteres*	*Firmicutes*	*Bacteroidetes*	*Spirochaetae*	*Proteobacteria*
0 (n = 8)	0	1.17	18.53	71.58	0.88	7.82
10 (n = 36)	0	0.82	19.07	74.12	0.95	4.78
	15	0.30	17.62	71.96	2.86	6.91
	30	0.63	19.03	69.98	2.51	7.59
	45	0.44	16.11	71.18	3.07	8.87
20 (n = 36)	0	1.79	18.40	65.57	4.89	8.98
	15	0.27	19.12	71.00	1.90	7.40
	30	0.20	21.06	63.76	4.34	10.11
	45	0.33	21.08	66.72	2.43	8.77
30 (n = 36)	0	0.38	22.72	68.08	1.38	7.27
	15	0.59	17.31	72.54	1.53	7.72
	30	0.91	21.50	64.55	3.65	9.09
	45	0.31	22.95	63.19	1.81	11.35
SEM		0.024	1.477	2.808	0.198	1.348
P value						
Green forage		0.507	0.458	0.275	0.451	0.246
Fattening duration		0.233	0.056	0.041	0.013	0.268
Green forage×fattening duration		0.711	0.609	0.931	0.259	0.862

At the order level (Table 4 and S6B Fig), *Clostridiales* was the second most abundant, and it exhibited a time-dependent increase (P = 0.004, R^2^ = 0.991). *Clostridiales* constituted 9.02 and 17.70% before and 30 days after fattening, respectively. *Bacteroidales* exhibited a significantly different abundancebased on the different fattening durations (P = 0.021). The *Selenomonadales* percentage exhibited a strong negative linear relationship with the fattening duration (P = 0.023, R^2^ = 0.955), with the highest percentage occurring in geese before fattening (P < 0.0001). The percentage of *Spirochaetales* in the cecal samples of geese before fattening was significantly lower than that of geese fattened for 10, 20, and 30 days (P = 0.001), but no significant regression with fattening duration was observed (P = 0.400, R^2^ = 0.359).

**Table 4:** Abundances of the major ordersbased on groups and fattening times.

Fattening duration	Green forage	*Clostridiales*	*Desulfovibrionales*	*Erysipelotrichales*	*Bacteroidales*	*Selenomonadales*	*Spirochaetales*
0 (n = 8)	0	10.59	9.06	1.26	69.63	8.76	0.69
10 (n = 36)	0	8.83	4.86	0.93	74.64	9.41	1.35
	15	10.00	5.13	1.80	74.42	5.39	3.26
	30	13.96	5.36	1.13	71.65	5.03	2.86
	45	11.68	8.82	0.72	71.19	4.38	3.22
20 (n = 36)	0	16.06	9.17	0.98	64.43	5.42	3.94
	15	12.80	5.43	1.01	71.33	6.95	2.48
	30	14.44	9.18	1.32	68.92	3.66	2.47
	45	18.32	8.44	1.11	65.21	3.33	3.59
30(n = 36)	0	15.07	3.80	0.74	74.67	4.17	1.54
	15	15.34	8.03	0.95	70.10	3.63	1.95
	30	18.27	7.15	0.65	68.63	2.08	3.21
	45	23.08	9.40	1.59	61.79	1.85	2.29
SEM		0.445	0.391	0.348	3.845	0.575	0.531
**P value**							
Green forage		0.103	0.204	0.921	0.227	0.024	0.355
Fattening duration		<0.0001	0.403	0.930	0.021	<0.0001	0.001
Green forage×fattening duration		0.751	0.583	0.466	0.870	0.110	0.204

The percentage of *Selenomonadales* exhibited a strong negative linear relationship with green forage supplementation (P = 0.029, R^2^ = 0.942), indicating that longer fattening durations resulted in lower *Selenomonadales* percentages in the cecal samples of geese fed a diet without green forage supplementation than in samples of those fed diets supplemented with 30 or 45% green forage (P = 0.024).

At the class level (Table 5 and S6C Fig), the most abundant microbial community was *Bacteroidia*, followed by *Clostridia*, *Deltaproteobacteria*, and *Negativicutes*. The *Negativicutes* percentage exhibited a strong negative linear relationship with fattening time (P = 0.023, R^2^ = 0.954), indicating that a longer fattening time resulted in a lower percentage of *Negativicutes*. However, the highest *Negativicutes* percentage was found in the cecal samples of geese fed a diet without green forage supplementation (P < 0.0001). The *Clostridia* percentage exhibited a strong positive linear relationship with fattening time (P = 0.004, R^2^ = 0.992), indicating that a longer fattening time resulted in a higher percentage of *Clostridia*, and the highest percentage was detected in the cecal samples of geese fed a diet with 45% green forage supplementation (P < 0.0001).

**Table 5:** Abundance of the major classesbased on green forage fed groups and fattening times.

Fattening time	Green forage	*Negativicutes*	*Deltaproteobacteria*	*Clostridia*	*Spirochaetes*	*Bacteroidia*	*Erysipelotrichia*
0 (n = 8)	0	8.50	6.92	8.97	0.90	73.30	1.02
10 (n = 36)	0	8.15	3.85	9.44	0.97	75.70	1.14
	15	5.46	6.31	10.80	2.90	72.92	1.27
	30	4.95	6.17	13.13	2.60	71.47	0.97
	45	3.91	8.11	11.64	3.12	72.17	0.67
20 (n = 36)	0	4.19	7.92	13.81	5.01	67.45	0.91
	15	4.95	5.42	13.44	1.93	71.95	0.82
	30	2.67	9.11	16.92	4.42	64.76	1.76
	45	4.17	7.06	16.28	2.50	68.19	1.05
30 (n = 36)	0	3.46	5.53	18.36	1.41	69.24	1.14
	15	3.18	7.21	13.43	1.55	73.47	0.91
	30	2.62	7.91	18.53	3.75	65.81	0.87
	45	1.52	10.74	20.15	1.84	64.05	1.45
SEM		0.096	1.312	1.493	0.387	2.683	0.273
P value							
Green forage		0.026	0.197	0.095	0.435	0.254	0.919
Fattening duration		<0.0001	0.404	<0.0001	0.013	0.019	0.932
Green forage×fattening duration		0.118	0.564	0.751	0.265	0.904	0.453

No significant linear relationship was detected between the percentage of *Spirochaetes* and *Bacteroidia* and fattening time in geese. However, the percentage of *Spirochaetes* from geese cecal samples collected before fattening was significantly lower than that of geese fattened for 10 and 20 days (P = 0.044 and P = 0.001, respectively). Furthermore, the percentage of *Bacteroidia* was higher in the cecal samples of geese before fattening and those fattened for 10 days (P = 0.022 and P = 0.032, respectively) than it was in samples from geese fattened for 20 and 30 days (P = 0.019 and P = 0.030, respectively).

The percentage of *Negativicutes* exhibited a strong negative linear relationship with green forage supplementation (P = 0.029, R^2^ = 0.942), and the percentage in the cecal samples of geese fed a diet without green forage was significantly higher than that found in samples from geese fed 30 or 45% green forage (P = 0.013 and P = 0.009, respectively).

At the family level (Table 6 and S6D Fig), the most abundant microbial community was *Bacteroidaceae*, followed by *Prevotellaceae*, *Ruminococcaceae*, *Desulfovibrionaceae*, *Rikenellaceae*, *Porphyromonadaceae*, *Veillonellaceae*, and *Acidaminococcaceae*. The *Prevotellaceae* percentage was significantly different among fattening durations (P < 0.0001), with highest percentage in cecal samples from geese collected before fattening. However, the *Prevotellaceae* percentage exhibited no linear correlation with fattening duration (P = 0.141). The *Porphyromonadaceae* and *Ruminococcaceae* percentages exhibited strong positive correlations with fattening duration (P = 0.045, R^2^ = 0.912 and P = 0.009, R^2^ = 0.982, respectively). The *Porphyromonadaceae* percentage from the cecal samples of geese fattened for 30 days was significantly higher than that of samples collected before (P = 0.001) or after fattening for 10 days (P = 0.014). The *Ruminococcaceae* percentage was lower in geese before or after fattening for 10 days (P < 0.0001 and P < 0.0001, respectively) than in those fattened for 20 and 30 days (P = 0.002 and P < 0.0001, respectively). The *Bacteroidaceae* percentage was lower in geese before fattening than it was in geese fattened for 10 and 30 days (P < 0.0001 and P = 0.0006, respectively). The *Acidaminococcaceae* percentage was higher in geese fattened for 10 days than it was in geese fattened for 20 and 30 days (P = 0.013 and P = 0.003, respectively). The *Veillonellaceae* percentage exhibited a strong negative linear relationship with fattening time (P = 0.044, R^2^ = 0.914), indicating that the highest percentage occurred in geese before fattening, while the lowest was observed in geese fattened for 30 days (P <0.0001). The microbial communities did not differ among the green forage supplementation groups.

**Table 6:** Abundance of the major familiesbased on green forage fed groups and fattening times.

Fattening time	Green forage	*Desulfovibrionaceae*	*Prevotellaceae*	*Porphyromonadaceae*	*Bacteroidaceae*	*Acidaminococcaceae*	*Ruminococcaceae*	*Rikenellaceae*	*Veillonellaceae*
0 (n = 8)	0	7.15	31.29	3.78^b^	30.09	2.76	7.82	5.05	5.98
10 (n = 36)	0	4.05	23.75	4.31^b^	42.94	3.68	8.03	3.80	4.86
	15	6.81	19.85	4.74^b^	38.48	3.27	9.00	7.49	2.59
	30	6.75	19.87	4.36^b^	40.96	2.18	10.39	5.71	3.13
	45	8.85	17.70	5.29^b^	39.66	2.46	9.34	6.91	1.80
20(n = 36)	0	9.18	22.27	5.61^b^	34.70	2.23	11.50	6.43	2.31
	15	5.88	30.01	4.92^b^	33.52	2.14	11.13	4.97	3.16
	30	10.31	20.01	4.88^b^	30.53	2.21	13.84	9.19	0.71
	45	7.72	19.39	4.46^b^	36.92	1.99	13.73	5.92	2.49
30 (n = 36)	0	5.98	15.10	11.98^a^	38.50	1.99	15.37	5.12	1.72
	15	7.78	20.97	3.81^b^	38.46	2.71	11.36	7.23	0.71
	30	8.92	15.11	6.00^b^	38.25	1.75	15.77	6.13	1.14
	45	11.73	12.27	5.40^b^	39.75	1.28	15.83	6.68	0.38
SEM		1.432	3.437	1.013	2.734	0.380	1.378	0.876	0.715
P value									
Green forage		0.186	0.323	0.067	0.817	0.153	0.245	0.214	0.258
Fattening duration		0.288	<0.0001	0.011	<0.0001	0.006	<0.0001	0.175	<0.0001
Green forage×fattening duration		0.449	0.907	0.031	0.971	0.585	0.912	0.086	0.326

At the genus level (Table 7 and S6E Fig), the most abundant microbial community was *Bacteroides*, followed by *Prevotella* and *Desulfovibrio*. The *Prevotella* percentage was highest in cecal samples collected from geese before fattening compared tothe examined durations (P < 0.0001). The percentage of *Bacteroides* was higher in samples collected from geese fattened for 10 or 30 days than it was in those collected from geese beforeor after fattening for 20 days (P < 0.0001). The percentage of *Oscillospira* was higher in geese fattened for 20 and 30 days than it was in geese before or after fattening for 10 days. The percentage of *Incertaesedis* was higher in geese fattened for 30 days than it was in geese fattened for the other durations (P = 0.006). The *Megamonas* percentage exhibited a strong negative relationship with fattening time (P = 0.047, R^2^ = 0.908), and a higher percentage was detected in geese before fattening than in geese at the other three fattening durations (P < 0.0001). Furthermore, a higher percentage of *Megamonas* was detected in geese fattened for 10 days than in those fattened for 20 and 30 days (P = 0.003 and P = 0.0004, respectively). The percentage of *Phascolarctobacterium* was higher in geese before or after fattening for 10 days than in geese fattened for 20 and 30 days (P = 0.004).

**Table 7:** Abundance of the major genera based on green forage fed groups and fattening times.

Fattening time	Green forage	*RC9gutgroup*	*Alistipes*	*Parabacteroides*	*Prevotella*	*Bacteroides*	*Incertaesedis*		*Desulfovibrio*	*Megamonas*	*Phascolarctobacterium*
0 (n = 8)	0	1.89	3.35	2.17	21.41	32.97	3.13		7.77	3.32^a^	3.04
10 (n = 36)	0	1.68	2.38	3.26	12.62	45.44	2.90		4.34	3.35^a^	3.93
	15	4.09	3.24	3.14	8.13	40.01	3.06		7.05	1.67^bc^	3.41
	30	2.41	3.32	2.67	9.10	43.71	3.63		7.08	2.00^b^	2.33
	45	2.73	4.16	3.44	5.80	41.72	2.70		9.26	0.97^c^	2.57
20 (n = 36)	0	3.78	2.36	2.78	12.93	35.74	3.05		9.09	1.06^bc^	2.30
	15	2.45	2.59	3.02	19.49	36.07	3.71		6.35	1.51^bc^	2.27
	30	3.82	4.91	2.97	8.66	31.17	3.41		10.38	0.54^c^	2.30
	45	2.35	3.64	3.27	8.69	39.23	2.94		8.13	1.42^bc^	2.11
30 (n = 36)	0	2.20	3.57	4.12	5.05	44.35	7.34		6.87	1.66^bc^	2.26
	15	3.29	3.84	2.58	6.52	39.27	3.39		7.93	0.60^c^	2.77
	30	2.71	3.68	3.29	4.52	40.63	5.11		9.52	0.90^c^	1.85
	45	4.03	3.18	3.07	0.72	44.18	4.36		13.25	0.39^c^	1.41
SEM		0.388	0.589	0.333	1.478	2.812	0.715		1.492	0.365	0.403
P value											
Green forage		0.827	0.250	0.524	0.394	0.525		0.450	0.142	0.042	0.159
Fattening duration		0.147	0.947	0.001	<0.0001	<0.0001		0.006	0.236	<0.0001	0.004
Green forage×fattening duration		0.422	0.484	0.549	0.913	0.866		0.404	0.532	0.038	0.637

The percentage of *Oscillospira* in the cecal samples of geese exhibited a strong positive linear relationship with green forage supplementation (P = 0.023, R^2^ = 0.954). The percentage of *Oscillospira* in the cecal samples of geese fed a diet without green forage was lower than that of geese fed 30 and 45% green forage (P = 0.009 and P = 0.002, respectively). The percentage of *Megamonas* was higher in geese fed a diet without green forage than it was in geese fed diets with 30 and 45% green forage supplementation (P = 0.024 and P = 0.008, respectively). The interaction between green forage and fattening duration suggested that short fattening time and lower green forage inclusion resulted in higher percentage of *Megamonas* (Table 7).

### 3.6. Fatty acid content in breast muscle of Wanxi white geese fed different level of green forage for different fattening duration

Linoleic acid (C18:2) was lower in geese before fattening as compared with other time points with or without green forage inclusion (P< 0.05, Table 8). Geese fed 15% green forage and fattening for 30 days exhibited higher C18:2 as compared with others (P< 0.05). Linolenic acid (C18:3) was higher in geese fed diet with 30–45% green forage inclusion and fattening for 20–30 days as compared with geese before fattening, fattening for 10 days, or fattening with 15% green forage inclusion in diet (P< 0.05, Table 8).

**Table 8:** Fatty acid content in geese fed different level of green forage for different fattening duration (%).

Fattening duration	Green forage	C16:0	C16:1	C18:0	C18:1	C18:2	C18:3	C20:3
0 (n = 8)	0%	20.09	1.01	18.72	26.78	17.28^d^	0.25^bc^	13.30
10 (n = 36)	0%	21.98	1.11	18.52	23.77	18.14^bc^	0.15^c^	13.04
	15%	20.42	1.05	18.89	25.31	17.59^cd^	0.20^bc^	13.15
	30%	22.36	1.26	17.27	25.04	17.87^bc^	0.28^bc^	12.53
	45%	22.11	1.04	17.84	24.11	18.65^ab^	0.30^bc^	12.47
20 (n = 36)	0%	23.07	1.21	16.73	25.62	18.10^bc^	0.36^b^	11.69
	15%	23.79	1.52	14.54	29.04	17.59^cd^	0.51^a^	9.75
	30%	21.63	1.28	16.80	25.80	18.60^ab^	0.41^ab^	12.21
	45%	22.90	1.49	16.20	26.30	18.14^bc^	0.48^a^	12.04
30 (n = 36)	0%	21.68	1.81	15.52	27.16	19.44^a^	0.35^b^	11.25
	15%	23.30	1.56	14.48	27.98	19.51^a^	0.49^a^	9.81
	30%	23.63	2.03	13.70	29.41	18.47^ab^	0.57^a^	9.34
	45%	23.37	1.88	14.23	28.35	18.88^ab^	0.55^a^	9.91
SEM		0.24	0.05	0.26	0.42	0.13	0.02	0.27
P		0.156	0.326	0.284	0.837	0.050	0.017	0.541

### 3.7. Canonical correspondence analysis (CCA) between microbiota and FA component in breast muscle

**Fig 6:**
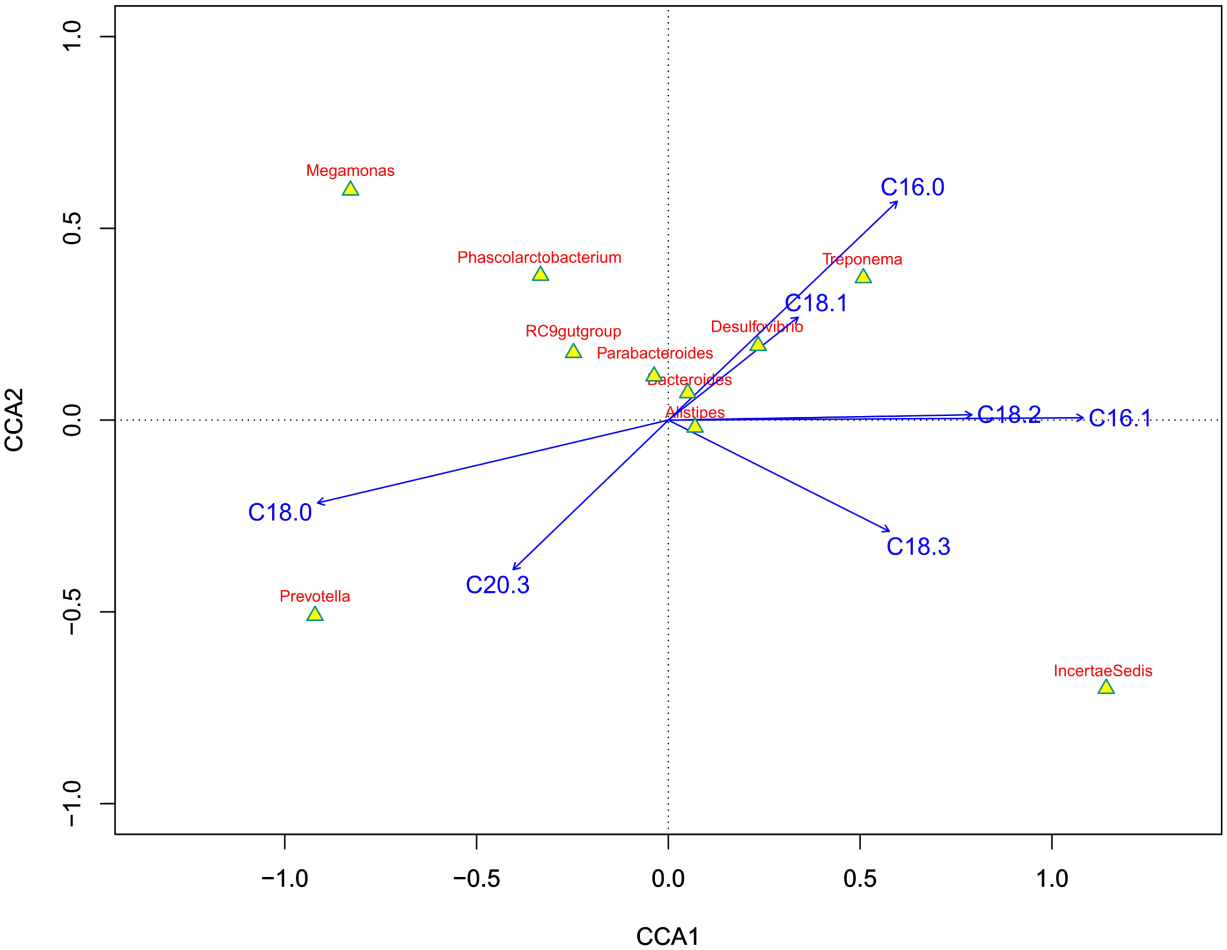
Canonical correspondence analysis (CCA) of the relationship between the relative abundance of bacteria (phylum level) and the percentage of muscle fatty acid (FA, environmental variables). Microbial phyla are shown as yellow triangles, and muscle FAs are shown as blue arrows. Microbial phyla can be vertically projected along the ray elongated from the blue arrows. The abundance was measured as the relative distance from the projection to the arrow, and the distance along the direction of the arrow was considered an increase (and vice versa). Acute and obtuse angles between the environmental variables and phyla were considered positive and negative correlations, respectively.

Canonical correspondence analysis (CCA) is a multivariate extension of weighted averaging ordination, which is a simple method used to arrange microbial communities based on environmental variables. CCA was used to rank microbial communities based on their importance in determining FA composition. The ordination diagram generated using CCA visualizes the main features of microbial community distribution based on FA variables as well as the importance of those variables. The CCA ordination diagram is constructed and interpreted as follows (Fig 6): the arrows represent the FA variables; the coordinates of the arrows are the values of the arrows on the two best synthetic gradients (axes 1 and 2 in Fig 6); the direction of the arrows represents the maximum change of the associated variables; the length of an arrow representing a FA variable is equal to the rate of change in the weighted averaging ordination, and is a measure of how much the microbial communities differ with regard to the FA variables.

Important FAs tend to be represented by longer arrows than less important ones. According to Fig 6, *Bacteroides* correlated with nearly all detected FAs, and the greatest correlations were associated with eicosatrienoic acid (C20:3), palmitic acid (C16:0), and oleic acid (C18:1). These results suggest that percentage of C20:3, C16:0, and C18:1 could be increased when geese are fattened 10–30 days. *Megamonas*, *Phascolarctobacterium*, RC9gutgroup, and *Parabacteroides* were all set near the reverse extension line of linolenic acid (C18:3), while others such as *Treponema*, *Desulfovibrio*, and *Prevotella* were set along the line of C18:1.

## 4. Discussion

In China, geese reproduction exhibits strong seasonal adaptation, and the birds usually lay eggs during winter and early spring. The eggsthen hatchin the spring, and goose fattening is performed when the birds areapproximately 60–70 days old duringthe summer. Geese are herbivorous, so green forage was suggested to be includedtoreduce feed conversion ratio and promote health [6]. In China, the most commonly available green forage during summer is white cabbage, and reports suggest that green forage could regulate intestinal microflora, thereby improving feed utilization [6,13].

Although no difference was observed in bacterial communities, our results indicated that green forage inclusion and fattening duration could numerically increase α-diversity and PD. These findings are highly in accordance with those of Wang et al. [14], whichreported that green forage or crude fiber could enrich intestinal microflora.

Ruminants can digest crude fiber because they possess ruminal and cecal microbes [15]. However, in monogastric animals, the digestion of cellulose and hemicelluloses depends solely on colon and cecal bacteria. Microbiota that can digest crude fiber mainly inhabit the cecum, which is the most important site for feed fermentation in geese. Volatile FAs in the cecum are the final products of carbohydrate decomposition. Furthermore, the activity of cecal microbiota in animals could be influenced by factors such as feed and age [9,10]. Green forage inclusion is thought to enhance microbial activity in the cecum, thus promoting cellulose digestion in geese.

The cecal microflora of geese was mainly influenced by the fattening duration, and this result is in consistent with the findings of Pourabedin et al. [10], which reported that gut microbiota variated in chickens at different ages. Ban-Tokuda et al. [16] suggested that the level of *Bacteroidetes* correlated with body weight and feed intake, and it decreased with fattening. This suggests that fattening geese for 10 days might result in higher feed intake than fattening for other durations. The members of *Firmicutes* and *Bacteroidetes* phyla dominate microbiota of animals [17,18], and lean animals are thought to have higher and lower levels of *Bacteroidetes* and *Firmicutes*, respectively, among gut microbes [17]. Although more polyunsaturated FAs are produced in geese fattened for a longer time [12], the meat production performance does not seem to be ameliorated based on the high percentages of *Bacteroidetes* from the ceca microbiota. Proper green forage inclusion (15%) might enhance fat deposition, whereas 30–45% inclusion could decrease fat deposition.

Inigo et al. [19] reported that rabbits fed a diet containing glycerol showed an increased *Clostridiales* percentage during fattening. Many studies reported that green forage inclusion could promote FA synthesis in animals [20, 21], this might suggest that the increased *Clostridiales* percentage could accelerate FA synthesis, especially that of UFAs. The *Selenomonadales* order belongs to the *Firmicutes* phylum, and studies in mice demonstrated that the abundance of *Firmicutes* is proportionate to obesity levels, high efficiency of FA absorption, and fat deposits [17,22–23]. This suggests that green forage inclusion could alleviate fat deposition in geese. Walugembe et al. [24] stated that broilers fed a high fiber diet showed an increase in the relative abundance of the *Selenomonadales* order, and this result differs greatly from the results of our experiment. Cecal microbial communities differ considerably among poultry breeds [24], and they are mainly responsible for fermentable nutrient availability and FA synthesis [14]. The decreased abundance of *Selenomonadales* (class *Negativicutes*) in geese fed diet with high percentage of green forage or subjected to fattening for long period indicates that this microbiota might play a minor role in FA synthesis, and *Helicobacter* and *Megamonas* genera exhibited increased abundance.

It is known that *Prevotella* degrades readily metabolizable carbohydrates, and it is more abundant in low-fiber diets [25]. These data are consistent with our experimental results in that a higher abundance of *Prevotellaceae* was detected in geese before than after fattening, and a lower percentage of UFA was detected during this stage than at other stages. Putative taxonomic identifications have linked *Bacteroidia* and *Porphyromonadaceae* (unclassified *Bacteroidales*) strains with C18:1 as well as *cis*-9 and *trans*-11 conjugated linoleic acid concentrations in the rumen of cattle and sheep [26, 27]. These identifications explain why the highest *Porphyromonadaceae* and *Ruminococcaceae* percentages were detected in geese fattened for 10–30 days, and this might indicate that appropriate fattening durations should be used for geese. Increased levels of *Bacteroidia*, *Porphyromonadaceae*, and *Ruminococcaceae* in ceca likely reflects the need for fiber digestion in geese. A higher fiber intake than usual might significantly increase the abundance of *Acidaminococcaceae* in wild turkeys [28], and this is in consistent with the results of our study in that a higher abundance of *Acidaminococcaceae* was detected in geese fed green forage or those that had been fattened.

*Bacteroides* and *Prevotella* were collectively numerically dominant in the rumen of cows [29], and these genera might be responsible for nutrient utilization. Fiber inclusion might inhibit nutrient utilization, thus decreasing *Prevotella* in the ceca of geese. As described by Ghaffarzadegan et al. [30], the addition of fibers from guar gum and pectincould decrease the cecal abundance of *Oscillospira* and an unclassified genus in the *Ruminococcaceae* family. Green forage inclusion could increase *Oscillospira* abundance, which might indicate that fiber type could modulate cecal microbiota composition.

An increased abundance of *Megamonas* was detected in broilers fed a high fiber diet [24], and these results arein contrast to our present results. Considering the function of the ceca in broilers and geese, it is clear that the ceca is very small in broilers and responsible for water absorption; however, it is important for fiber digestion in geese. Dietary fiber is usually limited to less than 5% in broilers, whereas more than 10% is suggested in geese.

*Phascolarctobacterium* plays a critical role in the dynamic balance of gut microbiota, and it could be responsible for the production of short-chain FAs [31]. Essential FAssuch as linoleic acid and alpha-linolenic acid cannot be synthesized de novo. Therefore, the lower content of short-chain FAs might indicate a higher level of long chain UFAs and a lower abundance of *Phascolarctobacterium* in geese fed 30–45% green forage.

## 5. Conclusion

This study demonstrated that green forage inclusion and thefattening duration increased the abundance of cecal microflora in geese. The bacterial community was mainly influenced by the fattening duration, and *Bacteroidales* and *Selenomonadales* orders and *Megamonas* and *Oscillospira* genera were affected by green forage inclusion levels. Finally, *Bacteroidales* might represent an intestinal promoter that improves UFA synthesis.

## Supporting information

S1 Fig Relative abundances (% of total sequences) of the most abundant bacterial phyla identified based on Ion Personal Genome Machine (PGM) sequencing of the cecal contents of geese fed diets with different levels of green forage for fattening at different durations. (JPG)

S2 Fig Relative abundances (% of total sequences) of the most abundant bacterial classes identified based on Ion Personal Genome Machine (PGM) sequencing of the cecal contents of geese fed diets with different levels of green forage for fattening at different durations. (JPG)

S3 Fig Relative abundances (% of total sequences) of the most abundant bacterial orders identified based on Ion Personal Genome Machine (PGM) sequencing of the cecal contents of geese fed diets with different levels of green forage for fattening at different durations. (JPG)

S4 Fig Relative abundances (% of total sequences) of the most abundant bacterial family identified based on Ion Personal Genome Machine (PGM) sequencing of the cecal contents of geese fed diets with different levels of green forage for fattening at different durations. (JPG)

S5 Fig Relative abundances (% of total sequences) of the most abundant bacterial genus identified based on Ion Personal Genome Machine (PGM) sequencing of the cecal contents of geese fed diets with different levels of green forage for fattening at different durations. (JPG)

S6 Fig Microbial community structures at each taxonomic level. Dendrogram illustrating bacterial community similarity based on(A) phylum, (B) order, (C) class, (D) family, and (E) genus.
(PDF)

S1 Table Major components and nutrition levels of the experimental diets. (XLS)

S2 Table Total number of reads and valid reads from the original FASTQ files associated with each sample. Valid reads (fragments) were obtained after short and mismatched sequences were removed. Valid ratio = Valid reads/Raw reads (%).
(XLS)

S3 Table Most abundant bacterial communities at phylum, class, order, family, and genus levels in geese fed diets with different levels of green forage for fattening at different durations. (XLSX)

## References

[1] Jørgensen H, Zhao X, Knudsen KEB, Eggum BO. The influence of dietary fibre source and level on the development of the gastrointestinal tract, digestibility and energy metabolism in broiler chickens. British Journal of Nutrition. 1996; 75:379–395. 8785212 10.1079/bjn19960141

[2] Iji P, Saki AA, Tivey D. Intestinal development and body growth of broiler chicks on diets supplemented with non-starch polysaccharides. Animal Feed Science and technology. 2001; 89:175–188.

[3] Roofchaei A, Rezaeipour V, Vatandour S, Zaefarian F. Influence of dietary carbohydrases, individually or in combination with phytase or an acidifier, on performance, gut morphology and microbial population in broiler chickens fed a wheat-based diet. Animal Nutrition. 2017; 10.1016/j.aninu.2017.12.001 PMC6407079 30899811

[4] Min BR, Solaiman S, Gurung N, McElhenney W. The effect of forage-based meat goat production systems on live performance, carcass traits and fatty acid composition of Kiko crossbred goats. Journal of Animal Nutrition. 2016; 1:1–11

[5] Lou Y, Liu H, Wang J, Sun Z. Determination and comparison of digestion kinetics of two fibre sources in geese (Anseris). South African Journal of Animal Science. 2010; 40:70–77.

[6] Yin H, Huang J. Effects of soybean meal replacement with fermented alfalfa meal on the growth performance, serum antioxidant functions, digestive enzyme activities, and cecal microflora of geese. Journal of Integrative Agriculture. 2016; 15:2077–2086.

[7] Adamski, M, Kuzniacka J. Effect of fattening with oats on the quality of fat content in White Koluda goose. XXII International Poultry Symposium PB WPSA “Science for poultry practice-poultry practice for science” Olsztyn 6–8 September 2010; 214–215.

[8] Gimeno A, Alami AA, Abecia L, de Vega A, Fondevila M, Castrillo C. Effect of type (barley vs. maize) and processing (grinding vs. dry rolling) of cereal on ruminal fermentation and microbiota of beef calves during the early fattening period. Animal Feed Science and Technology. 2015; 199:113–126.

[9] Simon K, Verwoolde MB, Zhang J, Smidt H, de Vries Reilingh G, Kemp B, et al Long-term effects of early life microbiota disturbance on adaptive immunity in laying hens. Poultry Science. 2016; 95:1543–1554. 10.3382/ps/pew088 26976906

[10] Pourabedin M, Guan L, Zhao X. Xylo-oligosaccharides and virginiamycin differentially modulate gut microbial composition in chickens. Microbiome. 2015; 3:15 10.1186/s40168-015-0079-4 25874109 PMC4396176

[11] Vasaï F, BrugirardRicaud K, Cauquil L, Daniel P, Peillod C, Gontier K, et al Lactobacillus sakei modulates mule duck microbiota in ileum and ceca during overfeeding. Poultry Science. 2014; 93:916–925. 10.3382/ps.2013-03497 24706969

[12] Chen X, Zhao N, Zhang Y, Geng Z. The fatty acid profile in muscles and expression of PPARα, FADS2 and ME1 genes in liver of Chinese Wanxi white geese in fattening period. Acta Veterinaria et ZootechnicaSinica. 2017; 48:1912–1919.

[13] Liu D, Zhou X, Zhao P, Gao M, Han H, Hu H. Effects of increasing non-fiber carbohydrate to neutral detergent fiber ratio on rumen fermentation and microbiota in goats. Journal of Integrative Agriculture. 2013; 12:319–326.

[14] Wang W, Cao J, Li J, Yang F, Li Z, Li L. Comparative analysis of the gastrointestinal microbial communities of bar-headed goose (Anser indicus) in different breeding patterns by high-throughput sequencing. Microbiological Research. 2016; 182:59–67. 10.1016/j.micres.2015.10.003 26686614

[15] Mohammadzadeh H, Yáñez-Ruiz DR, Martínez-Fernandez G, Abecia L. Molecular comparative assessment of the microbial ecosystem in rumen and faeces of goats fed alfalfa hay alone or combined with oats. Anaerobe. 2014; 29:52–58. 10.1016/j.anaerobe.2013.11.012 24333680

[16] Ban-Tokuda T, Maekawa S, Miwa T, Ohkawara S, Matsui H. Changes in faecal bacteria during fattening in finishing swine. Anaerobe. 2017; 47:188–193. 10.1016/j.anaerobe.2017.06.006 28610999

[17] Ley RE, Backhed F, Turnbaugh P, Lozupone CA, Knight RD, Gordon JI. Obesity alters gut microbial ecology, Proceedings of the National Academy of Sciences of the United States of America. 2005; 102:11070–11075. 10.1073/pnas.0504978102 16033867 PMC1176910

[18] Pedersen R, Ingerslev HC, Sturek M, Alloosh M, Cirera S, Christoffersen BO, et al Characterisation of gut microbiota in Ossabaw and Gottingen minipigs as models of obesity and metabolic syndrome, PLoS ONE. 2013; 8:e56612 10.1371/journal.pone.0056612 23437186 PMC3577853

[19] Inigo MA, Blas JCD, Cachaldora P, Garciarebollar P. Effect of starch substitution with crude glycerol on growing rabbit and lactating doe performance. World Rabbit Science. 2011; 19:67–74.

[20] Dhiman TR, Hagos SA, Walters JL, Tamminga S. Conjugated linoleic acid (CLA) and omega fatty acids in mild from cows fed green chopped forage. Journal of Dairy Science. 2005; 88: suppl 276.

[21] Mustafa AF and Baurhoo B. Evaluation of dried vegetable residues for poultry: III Effects of feeding cabbage leaf residues on laying performance, egg quality, and apparent total tract digestibility. Journal of Applied Poultry Research. 2017; 10.3382/ps/pew290 27587726

[22] Bäckhed F, Ding H, Wang T, Hooper LV, Koh GY, Nagy A, et al The gut microbiota as an environmental factor that regulates fat storage. Proceedings of the National Academy of Sciences of the United States of America. 2004; 101: 15718–15723. 10.1073/pnas.0407076101 15505215 PMC524219

[23] Turnbaugh PJ, Ley RE, Mahowald MA, Magrini V, Mardis ER, Gordon JI. An obesity-associated gut microbiome with increased capacity for energy harvest. Nature. 2006; 444: 1027–1031. 10.1038/nature05414 17183312

[24] Walugembe M., Hsieh JCF, Koszewski NJ, Lamont SJ, Persia ME, Rothschild MF. Effects of dietary fiber on cecal short-chain fatty acid and cecal microbiota of broiler and laying-hen chicks. Poultry Science. 2015; 94:2351–2359. 10.3382/ps/pev242 26316341

[25] Pitta DW, Pinchak W, Dowd S, Osterstock J, Gontcharova V, Youn E, et al Rumen bacterial diversity dynamics associated with changing from bermudagrass hay to grazed winter wheat diets. Microbial Ecology.2010; 59:511–522. 10.1007/s00248-009-9609-6 20037795

[26] Huws SA, Kim EJ, Lee MRF, Scott MB, Tweed JKS, Pinloche E, et al As yet uncultured bacteria phylogenetically classified as Prevotella, Lachnospiraceae incertae sedis and unclassified Bacteroidales, Clostridiales and Ruminococcaceae may play a predominant role in ruminal biohydrogenation. Environmental Microbiology. 2011; 13:1500–1512. 10.1111/j.1462-2920.2011.02452.x 21418494

[27] Castro-Carrera T, Toral PG, Frutos P, McEwan NR, Hervas G, Abecia L, et al Rumen bacterial community evaluated by 454 pyrosequencing and terminal restriction fragment length polymorphism analyses in dairy sheep fed marine algae. Journal of Dairy Science. 2013; 97:1661–1669. 10.3168/jds.2013-7243 24440247

[28] Scupham AJ, Patton TG, Bent E, Bayles DO. Comparison of the cecal microbiota of domestic and wild turkeys. Microbial Ecology. 2008; 56:322–331 10.1007/s00248-007-9349-4 18183454

[29] Stevenson DM, Weimer PJ. Dominance of Prevotella and low abundance of classical ruminal bacterial species in the bovine rumen revealed by relative quantification real-time PCR. Applied Microbiology and Biotechnology. 2007; 75:165–174. 10.1007/s00253-006-0802-y 17235560

[30] Ghaffarzadegan T, Marungruang N, Fak F, Nyman M. Molecular properties of guar gum and pectin modify cecal bile acids, microbiota, and plasma lipopolysaccharide-binding protein in rats. PLoS ONE. 2016; 11:e0157427 10.1371/journal.pone.0157427 27315087 PMC4912110

[31] Wu F, Guo X, Zhang J, Zhang M, Ou Z, Peng Y. Phascolarctobacterium faecium abundant colonization in human gastrointestinal tract. Experimental and Therapeutic Medicine. 2017; 14:3122–3126. 10.3892/etm.2017.4878 28912861 PMC5585883

