# Supplementary figures and images for: Green forage and fattening duration differentially modulate cecal microbiome of Wanxi white geese
Source: PLoS One. 2018 Sep 25;13(9):e0204210. doi: 10.1371/journal.pone.0204210 (PMC6155509; doi:10.1371/journal.pone.0204210)

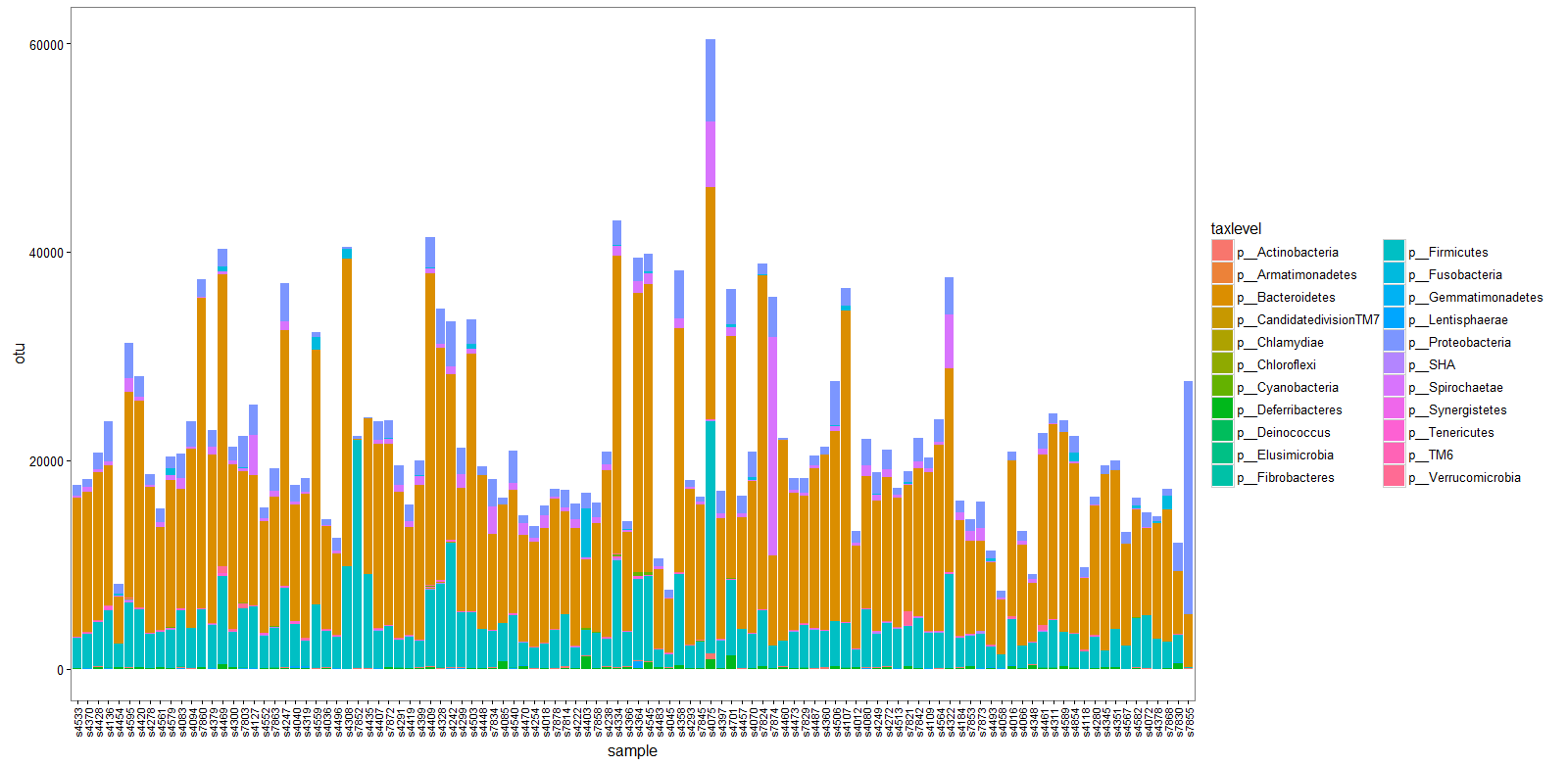

Supplement: S1 Fig — (JPG) [file pone.0204210.s001.jpg]

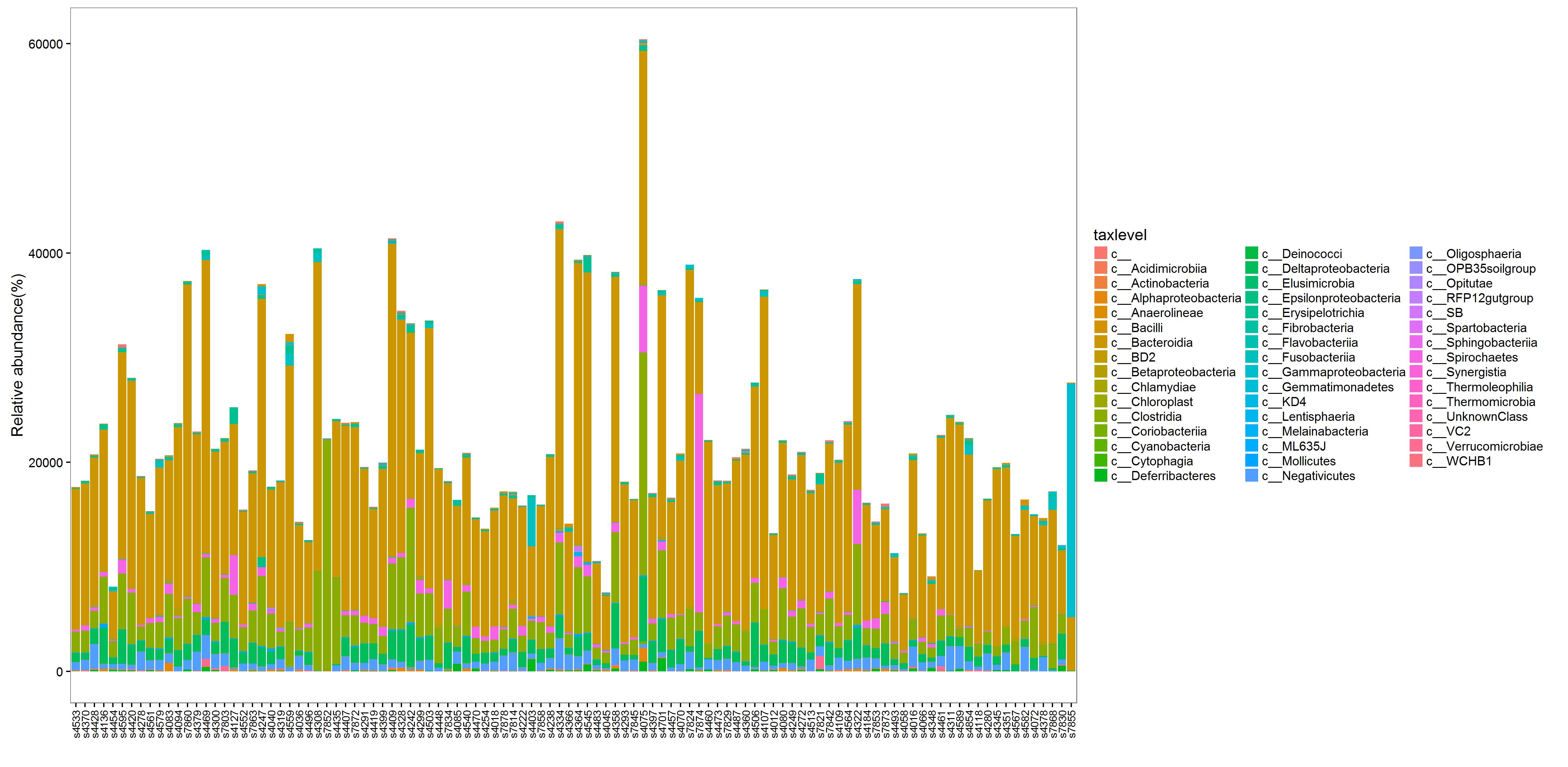

Supplement: S2 Fig — (JPG) [file pone.0204210.s002.jpg]

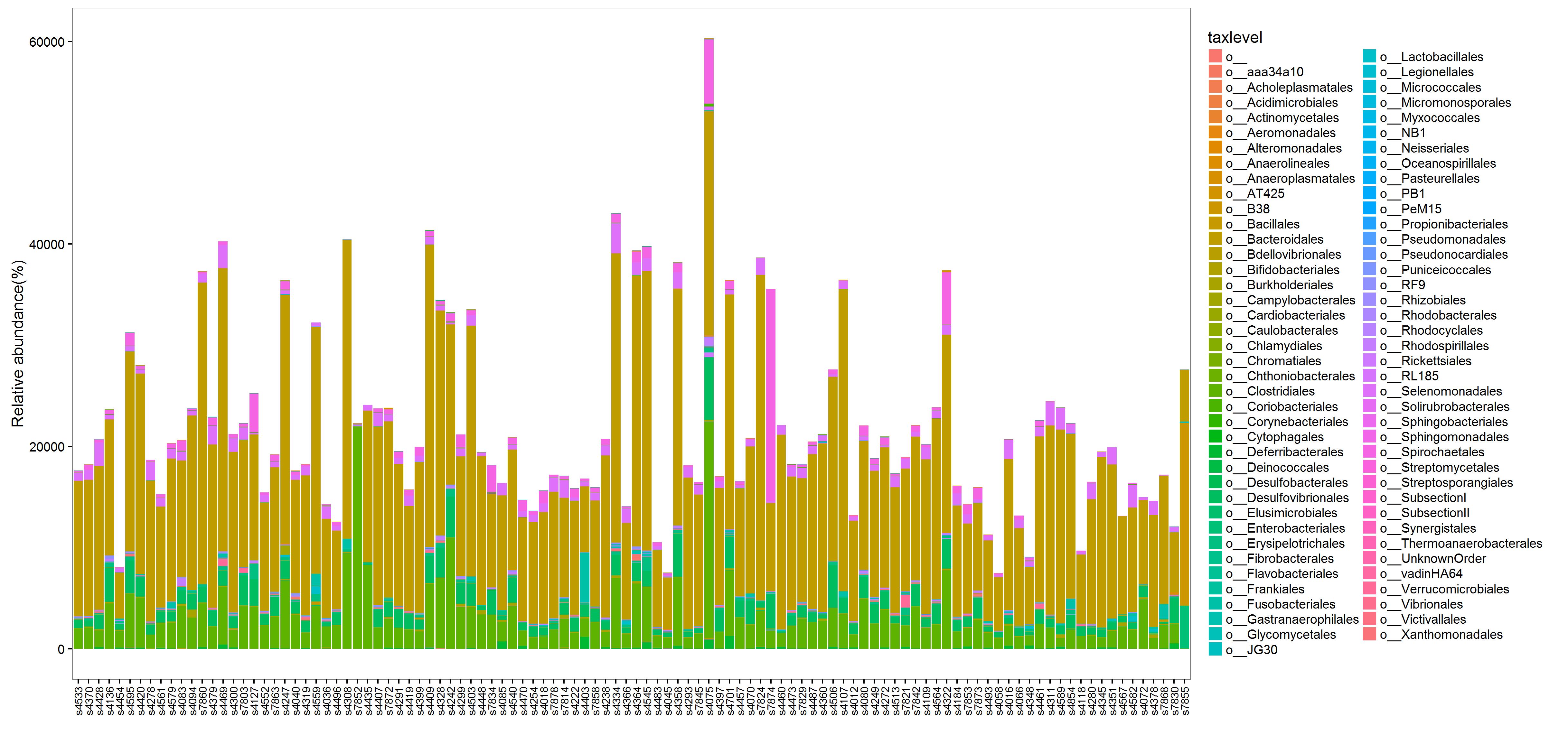

Supplement: S3 Fig — (JPG) [file pone.0204210.s003.jpg]

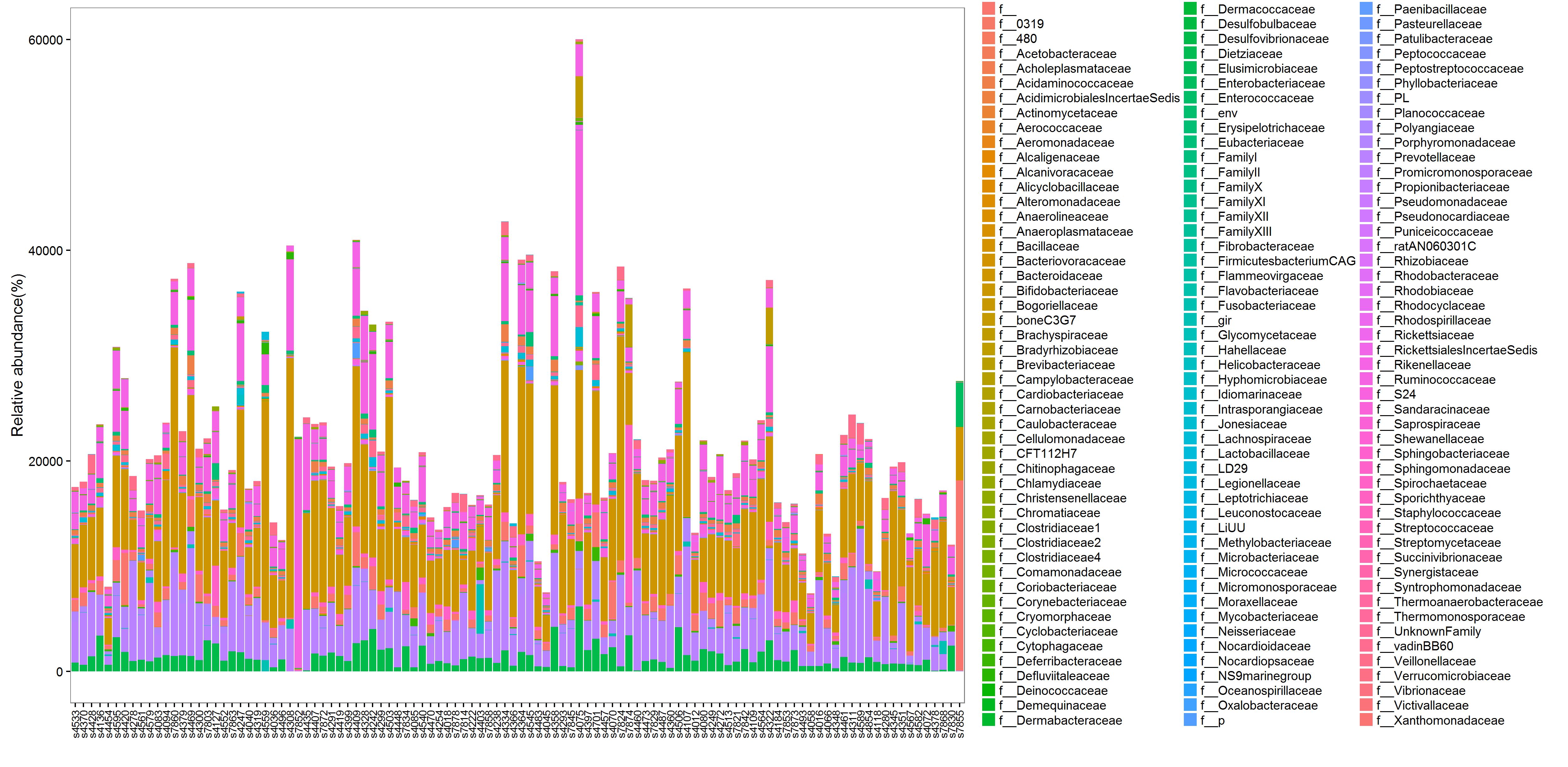

Supplement: S4 Fig — (JPG) [file pone.0204210.s004.jpg]

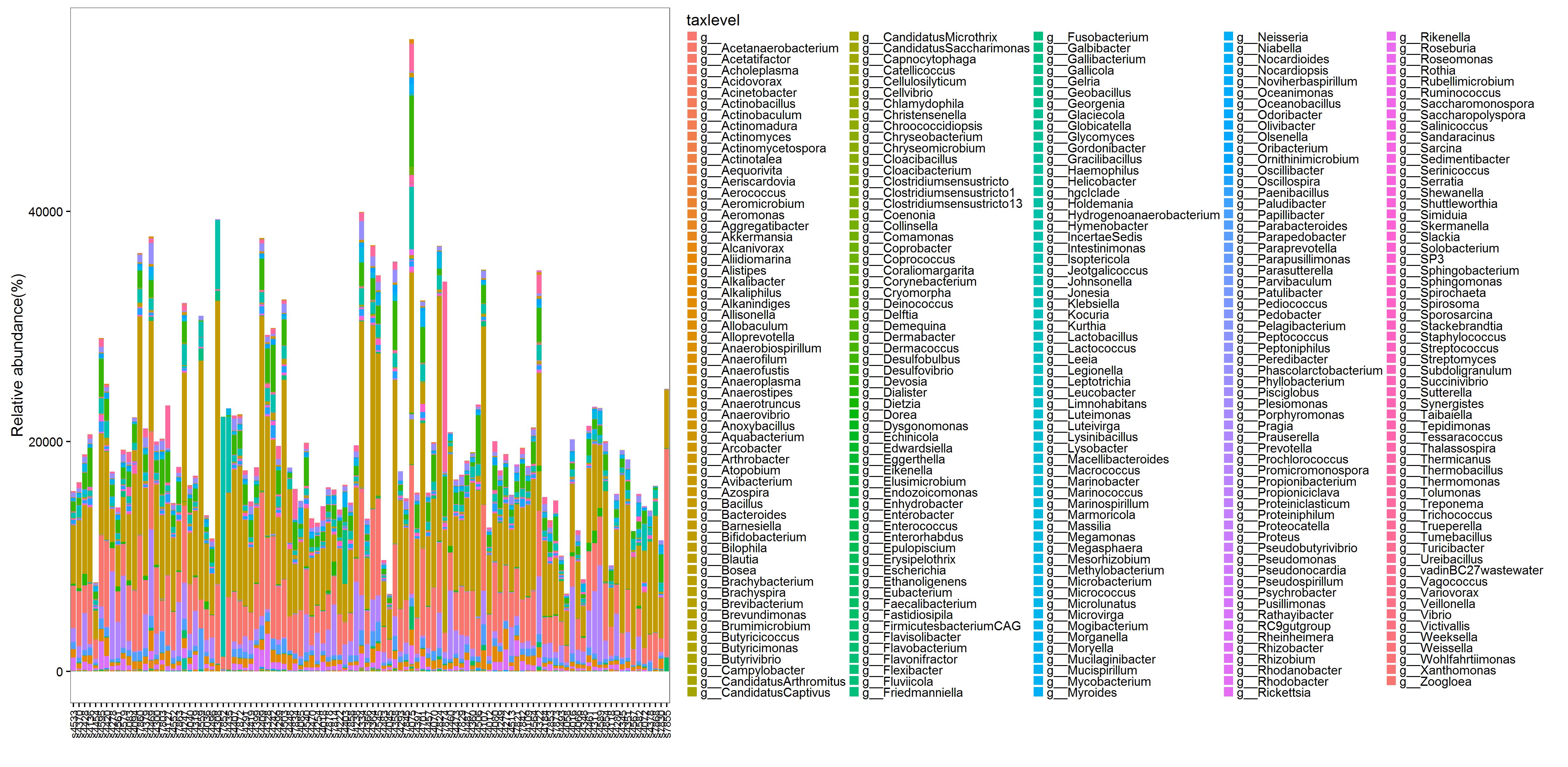

Supplement: S5 Fig — (JPG) [file pone.0204210.s005.jpg]

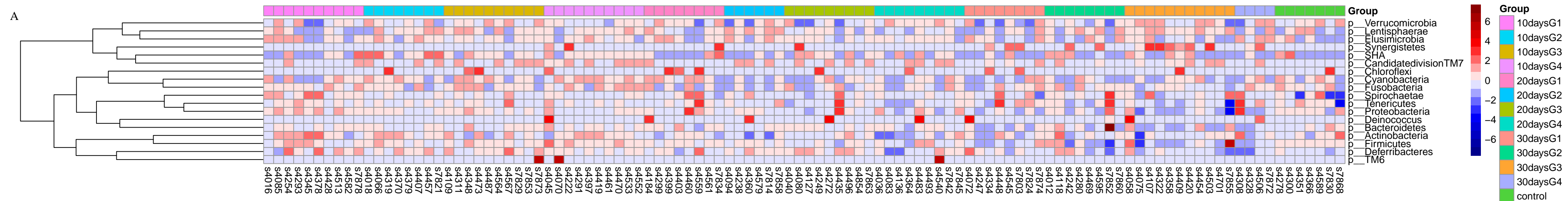

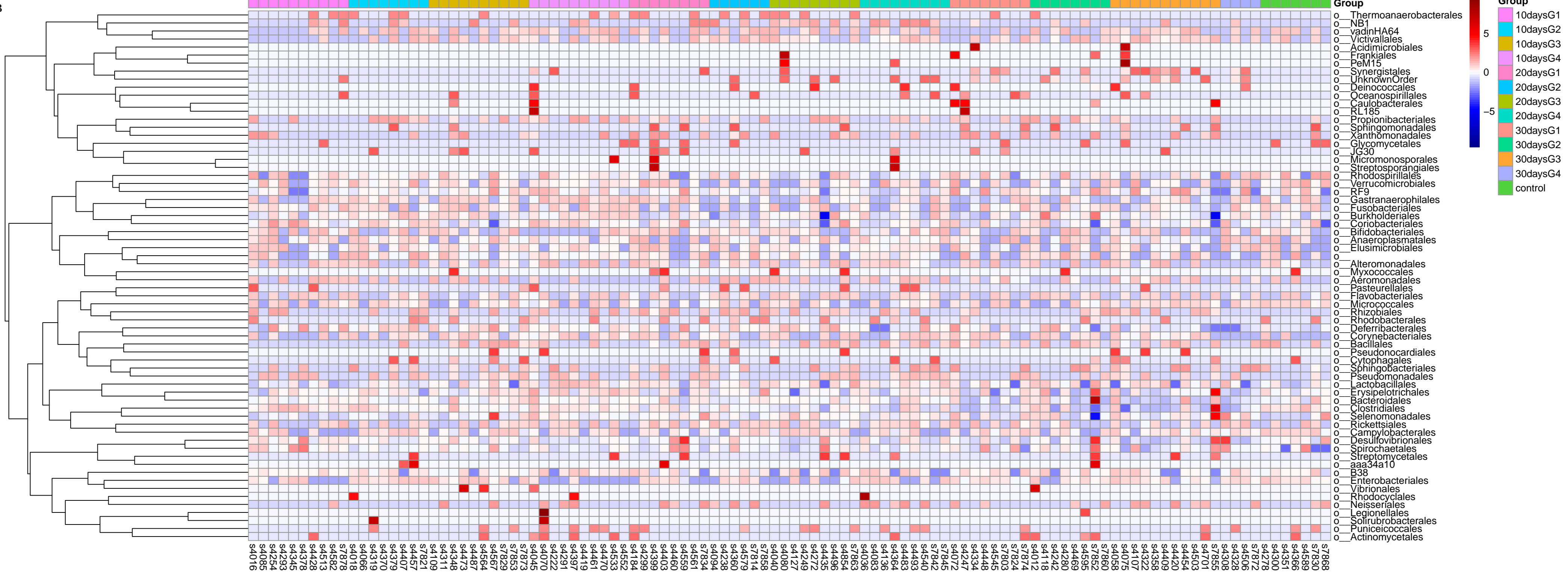

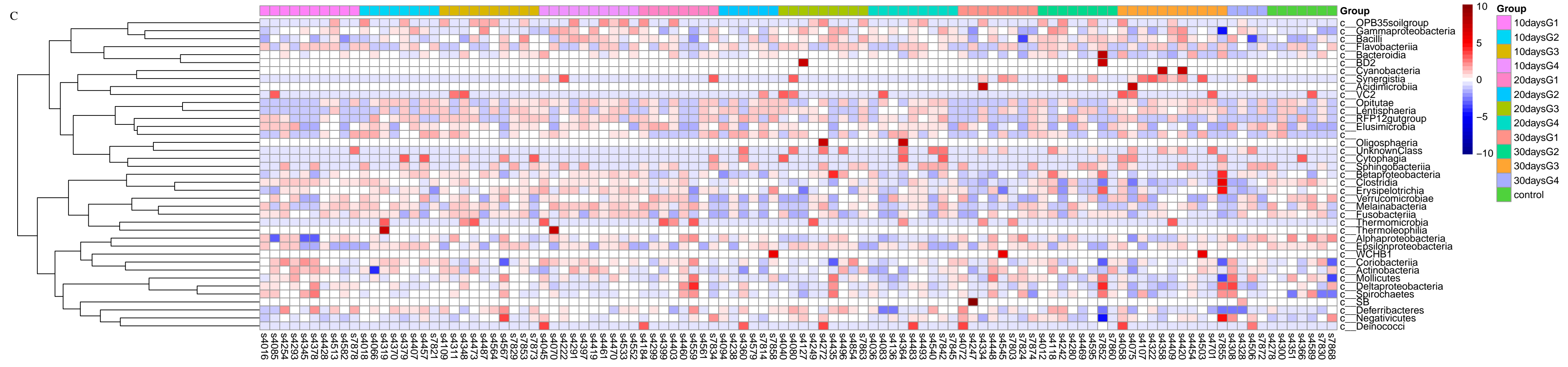

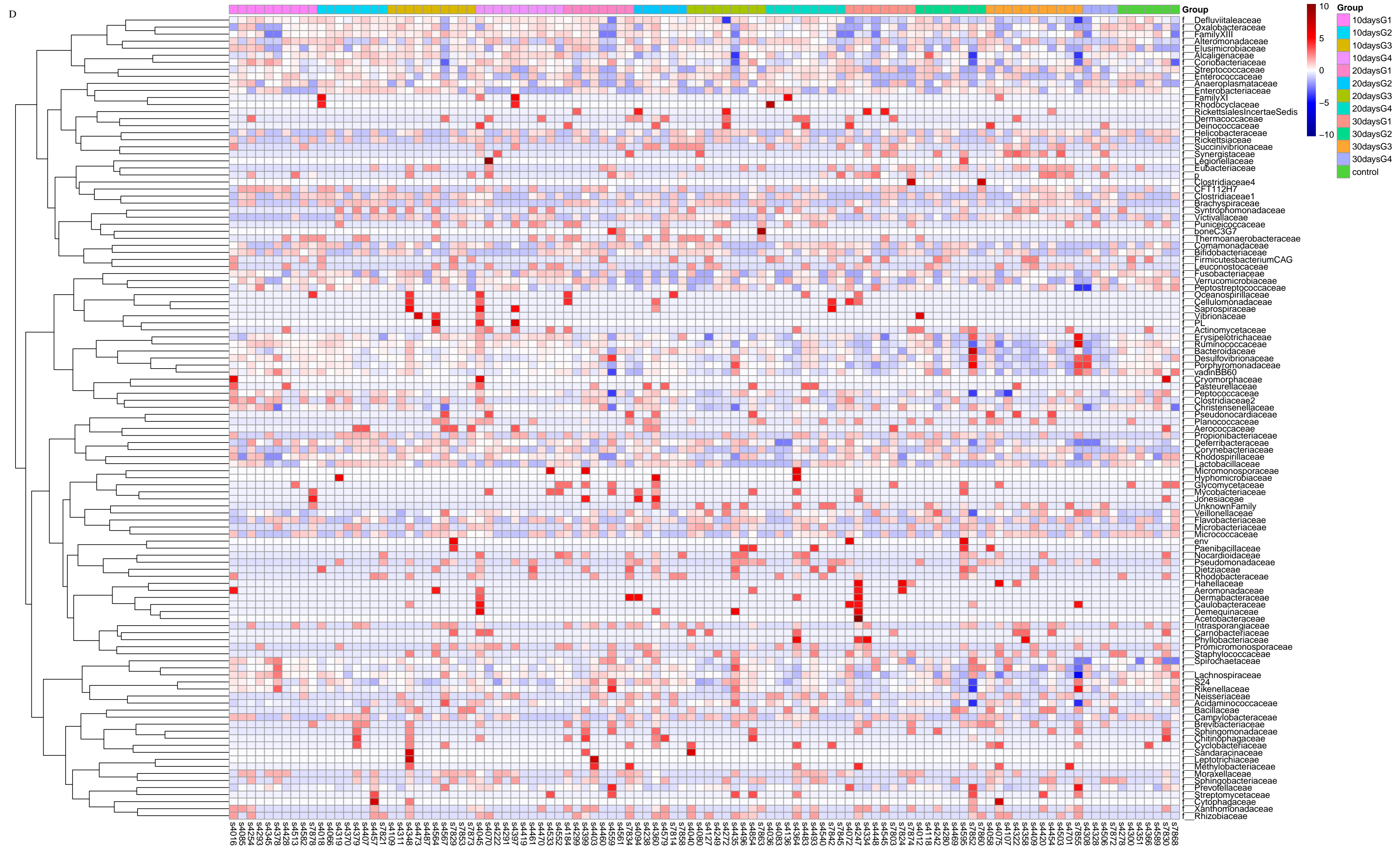

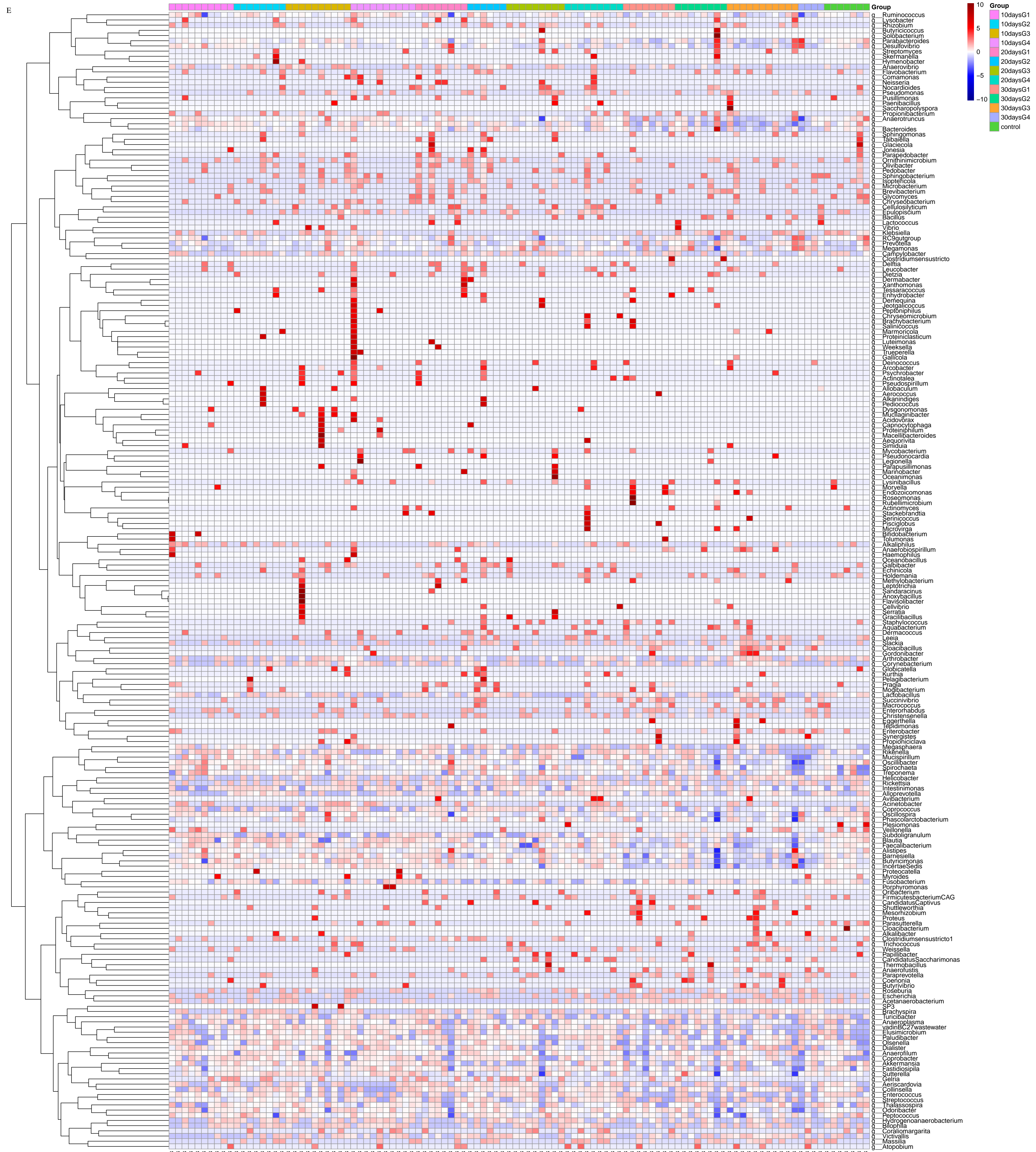

Supplement: S6 Fig — Dendrogram illustrating bacterial community similarity based on(A) phylum, (B) order, (C) class, (D) family, and (E) genus. (PDF) [file pone.0204210.s006.pdf]
